# Porcine Endogenous Retroviruses in Xenotransplantation—Molecular Aspects

**DOI:** 10.3390/v6052062

**Published:** 2014-05-13

**Authors:** Magdalena C. Kimsa, Barbara Strzalka-Mrozik, Malgorzata W. Kimsa, Joanna Gola, Peter Nicholson, Krzysztof Lopata, Urszula Mazurek

**Affiliations:** 1 Department of Food and Nutrition, Medical University of Silesia, Jednosci 8, 41-200 Sosnowiec, Poland; 2 Department of Molecular Biology, Medical University of Silesia, Jednosci 8, 41-200 Sosnowiec, Poland; E-Mails: bstrzalka@sum.edu.pl (B.S.-M.); gosiakimsa@gmail.com (M.W.K.); jgola@sum.edu.pl (J.G.); nicholson@wp.pl (P.N.); biolmolfarm@sum.edu.pl (K.L.); umazurek@sum.edu.pl (U.M.)

**Keywords:** porcine endogenous retroviruses, xenotransplantation, molecular techniques, detection

## Abstract

In the context of the shortage of organs and other tissues for use in human transplantation, xenotransplantation procedures with material taken from pigs have come under increased consideration. However, there are unclear consequences of the potential transmission of porcine pathogens to humans. Of particular concern are porcine endogenous retroviruses (PERVs). Three subtypes of PERV have been identified, of which PERV-A and PERV-B have the ability to infect human cells *in vitro*. The PERV-C subtype does not show this ability but recombinant PERV-A/C forms have demonstrated infectivity in human cells. In view of the risk presented by these observations, the International Xenotransplantation Association recently indicated the existence of four strategies to prevent transmission of PERVs. This article focuses on the molecular aspects of PERV infection in xenotransplantation and reviews the techniques available for the detection of PERV DNA, RNA, reverse transcriptase activity and proteins, and anti-PERV antibodies to enable carrying out these recommendations. These methods could be used to evaluate the risk of PERV transmission in human recipients, enhance the effectiveness and reliability of monitoring procedures, and stimulate discussion on the development of improved, more sensitive methods for the detection of PERVs in the future.

## 1. Introduction

According to the United States Public Health Service, xenotransplantation includes any procedure that involves the transplantation, implantation, or infusion into a human recipient of live cells, tissues, or organs from a nonhuman animal source or human body fluids, cells, tissues, or organs that have had *ex vivo* contact with live nonhuman animal cells, tissues, or organs [1]. The pig has been considered a potential source animal for xenotransplantation materials because of the comparable sizes of human and porcine organs, the similar physiological parameters of the two species, the ease of breeding pigs and the significant phylogenetic distance between pigs and humans, which reduces the risk of transmission of viral infections. Porcine endogenous retroviruses (PERVs) represent one of several types of viruses found in pigs that might be transmitted to humans through xenotransplantation [2,3,4,5,6,7].

### 1.1. Xenotransplantation Trials

Porcine materials such as livers, splenic or kidney perfusion *ex vivo*, fetal pig neural cells, porcine islets, corneas, and skin have been used in previous studies to treat different human diseases [8,9,10,11,12]. In addition, porcine heart valves have been widely used for many years in replacement cardiac valve surgery [13]. However, nonliving animal biological materials are classified as medical devices, drugs, or biological products, but not as xenotransplantation products. A few xenotransplantation clinical trials, such as the investigation of the safety and effectiveness of DIABECELL^®^ (immunoprotected alginate-encapsulated porcine islets for xenotransplantation), have been conducted in patients with type 1 diabetes mellitus [14]. Retrospective studies have also been conducted to assess possible PERV infection in human xenograft recipients [15,16,17,18,19,20,21]. Paradis *et al*. [17] collected peripheral blood mononuclear cells (PBMCs) and serum samples from 160 patients who underwent different xenotransplantation procedures, such as extracorporeal liver, splenic or kidney perfusion, pancreatic islet cell transplantation, and skin xenografts. While 81% of those samples were PERV DNA-negative, some samples were found to be positive for the presence of pig centromeric or mitochondrial DNA, indicating microchimerism. No PERV RNA was found in the serum or saliva. Similar results were obtained in other studies, suggesting a lack of PERV infection in patients exposed to various porcine materials, including pig islets, skin grafts, livers, kidney and splenic perfusions, and heart valves [13,15,16,18,19,20]. However, the ability of PERVs to infect human cells *in vitro* has led to the development of diagnostic tools to detect viral infection in patients exposed to pig cells and tissues [2,22,23]. The similarity of PERV to other members of the γ-retrovirus genus might also suggest the risk of developing diseases associated with PERV infection, such as tumors, leukemia, and neurodegeneration [24,25,26,27]. Moreover, the transmission of various cross-species pathogens, including human immunodeficiency virus type 1 (HIV-1), influenza A virus subtype H1N1, and West Nile virus infections, is unpredictable and can lead to the development of different diseases [28,29,30,31,32,33]. Thus, the risk and benefits to individual patients and society as a whole should be taken into account in discussing xenotransplantation safety. One of the problems is that xenotransplantation products can insert pathogens from the donor animal into the organ recipient, thereby potentially spreading infectious diseases to the general population. On the other hand, it should be noted that xenotransplantation can often be a life-saving procedure [34].

### 1.2. The Structure, Tropisms and Subtypes of PERVs

PERVs belong to the genus *Gammaretrovirus*, and as with the genomes of all retroviruses, the PERV genome is constructed of three genes: group-specific antigen (*gag*), polymerase (*pol*) and envelope (*env*). At the DNA provirus stage, these genes are bounded by noncoding terminal repeat sections known as long terminal repeats (LTR), which contain promoter, enhancer, and regulatory elements. The *gag* gene encodes structural proteins, which comprise the capsid, nucleocapsid and matrix; the *pol* gene encodes reverse transcriptase, ribonuclease H, integrase, and protease; and the *env* gene encodes the transmembrane envelope protein (TM) and surface envelope protein (SU). The region that encodes the SU contains a receptor-binding domain. Within this domain, two variable regions that determine the tropism and subtype of the PERVs can be distinguished: variable regions A and B (VRA, VRB), as well as a third, proline-rich region also known to be essential for receptor binding [35,36,37,38]. 

Two subtypes of PERVs, PERV‑A and PERV‑B, are present in all pigs [27,39]. These two subtypes are polytropic and are able to infect human cells in addition to pig cells. A third subtype, PERV‑C, which is present in many but not all pigs, is an ecotropic virus—one that occurs and replicates only in porcine cells [39,40]. However, PERV‑A can recombine with PERV‑C, and these recombinant viruses (PERV-A/C) have the ability to infect human cells [36,41,42,43,44].

### 1.3. PERVs and Their Potential to Cause Xenozoonotic Disease

PERVs are integrated into the porcine genome and inherited as Mendelian traits. The expression of PERVs may differ, depending on the breed of pig and the tissue [45,46,47,48,49,50,51,52,53], but the PERV DNA copy number in the whole organism is about 50 copies per haploid genome [54,55]. Moreover, there are variations in PERV integration sites among breeds [56,57,58,59]. Groenen *et al.* [59] analyzed the genome sequence of a domestic Duroc pig and compared it with the genomes of wild and Europe and Asia domestic pigs. The authors identified 20 almost intact PERV γ1 *loci* and four β-retroviral PERVs, but with defects in the *gag*, *pol*, or *env*, indicating that these proviruses are not replicable. Moreover, these *loci* were different in the studied pigs, which might suggest considerable PERV polymorphisms. Endogenous retroviruses are proviruses integrated into the germ line of the host and inherited by the offspring. PERVs can be activated to emerge as potentially infectious virus particles; therefore, the existence of PERVs in exogenous form has been proposed, and a PERV-A/C recombinant, which appears to exist *in vivo*, has been isolated from PBMCs but has not been found in the germ line of the same individuals [60,61,62]. Martin *et al.* [63] demonstrated the presence of the recombinant PERV-A/C provirus in the genome of some porcine cells in some organisms. Some endogenous retroviruses can induce diseases, but they are generally nonpathogenic in their original hosts. Moreover, many endogenous proviral elements are transcriptionally silent or defective, carrying deletions or point mutations, and are thus incapable of producing an infectious virus [64,65]. However, some gammaretroviridae, such as feline leukemia virus, murine leukemia virus, gibbon ape leukemia virus, and koala retrovirus induce leukemia and immunodeficiency in the infected host [26]. PERVs are not known to cause disease, although a recent work reported an increased incidence rate of PERV-A/C viraemia in pigs suffering from clinical conditions including diarrhea, wasting, and respiratory disease compared to healthy pigs [66]. In addition, Dieckhoff *et al.* [67] detected elevated PERV expression in melanomas of Munich miniature swine Troll and pulmonary metastasis-derived melanoma cell cultures. There are many examples of trans-species transmissions of retroviruses [26], and the possibility of PERV-related disease occurring in human xenotransplant recipients must be taken seriously. PERVs have been shown to be able to infect human cells *in vitro* [3]; however, this phenomenon has not been observed *in vivo* [15,16,17,18,19,20,21,68]. PERV transmission detected in mice *in vivo* [69,70,71,72] is an artifact of microchimerism or pseudotyping with murine endogenous retroviruses. Murine cells do not have functional receptors for PERV [73,74,75,76]. While most PERV proviruses are unable to produce complete viruses, their expression of genetic material does allow some to produce replication-competent viruses [38]. In addition, while PERV-A/C recombinants have been shown to exhibit human tropism [42], the region in the SU that alters the binding and infectivity of PERV-C itself differs by only nine residues from the analogous region of PERV-A [37]; therefore, it could potentially undergo mutation and selection to a human-tropic form [77].

### 1.4. The Need to Screen for PERVs in Xenotransplantation

PERV DNA cannot be completely eliminated from materials used in xenotransplantation [78,79]. It is also difficult to eliminate PERV by designated pathogen-free pig breeding, as their presence in the host genome means they are inherited by the offspring. Thus the need to monitor transplant recipients for PERV infection has long been recognized [80]. Moreover, there is a need to look for virus-human junction fragments to provide unambiguous evidence of infection of human cells. Moalic *et al.* [81,82], by cloning and mapping PERV integration sites in infected human embryonic kidney 293 cells (HEK293), revealed an integration preferences of the PERV DNA genome near the transcriptional start sites and CpG islands of transcriptional active genes in the chromosomes, similar to murine leukemia virus. These authors also revealed 224 hot spots in the human genome [82]. In fact, it is important that screening for PERVs be carried out in both donors and recipients using sensitive and specific methods, and that it be carried out at the genome, transcriptome, and proteome stages. 

Against the background of growing potential for the use of a variety of porcine tissues in xenotransplantation, the International Xenotransplantation Association has specified conditions for undertaking clinical trials of porcine islet products in type 1 diabetes in terms of strategies to prevent the transmission of PERVs. However, the effect of PERV transmission to humans remains unclear [5].

Our efforts focused on the molecular aspects of PERV infection in xenotransplantation and a review of the techniques available for carrying out the International Xenotransplantation Association recommendations. The methods described in this article could be used to evaluate the risk of PERV transmission in human recipients, enhance the effectiveness and reliability of monitoring procedures, and stimulate discussion regarding the development of improved, more sensitive methods of detecting PERVs in the future.

## 2. The Four Strategies to Prevent Transmission of PERVs

The International Xenotransplantation Association has indicated the existence of four strategies to prevent the transmission of PERVs.These recommendations are as follows: (1) careful screening of the source pig herd for PERVs; (2) selection of pigs that exhibit low-level expressions of PERV-A and PERV-B; (3) selection of pigs that do not contain PERV-C in their germ line, to prevent recombination with PERV-A; and (4) screening of xenotransplant recipients for PERV transmission using assays that are sufficiently sensitive to enable differentiation between transmission and chimerism [1,68,83].

### 2.1. Careful Screening of the Source Pig Herd for PERVs

As with all retroviruses, PERVs use RNA as their genetic material. DNA is used in the retrovirus replication cycle, and it can be integrated into the host DNA as a provirus. Careful screening of the source pig herd for PERVs therefore includes analysis of both DNA and RNA. 

#### 2.1.1. Qualitative Analysis of PERV DNA

Detection of the PERV provirus genome can be achieved by the use of polymerase chain reaction (PCR), using primers that are complementary to a variety of PERV DNA sequences.

The first group is comprised of primers complementary to the conservative PERV genes *gag* and *pol*. Paradis *et al.* [17] and Sypniewski *et al.* [47] used PCR primers situated in a highly conserved region of the PERV genome, the *gag* sequence, to detect all PERV types in both human and porcine samples, respectively. In addition, Prabha and Verghese [49,84], with the use of PCR for PERV DNA and reverse transcriptase RT-PCR for PERV RNA analyses, found PERV-specific *gag* sequences in aortic valve, pulmonary valve, and heart muscle samples obtained from fresh porcine xenograft tissue. Kim *et al.* [85] detected PERV *gag* sequences in porcine cells by PCR to assess the usefulness of the one-step extraction method compared to the phenol extraction method, whereas Wang *et al.* [86] assessed the safety of a bioartificial liver support system. In turn, Wynyard *et al.* [87] used PCR to detect PERV *pol* sequences in porcine blood samples and multiplex PCR to detect PERV *pol* sequences, a pig cell marker for the determination of microchimerism and an internal amplification control in human blood samples obtained from DIABECELL^®^ recipients. Li *et al.* [23] used both primers specific to *gag* and *pol* sequences to test cells for the presence of PERV by PCR and RT-PCR. 

Detection of these genes in the test material indicates the presence of PERVs, but it does not indicate which subtype of the virus is present. Generally, it answers the question as to whether the material being tested contains genetic material of PERV origin [23,47,49,84,85,86,87,88]. The second group consists of primers complementary to *env*, a gene that is characterized by a large degree of variability. Use of these primers enables identification of the virus subtype [35,40,89]. Akiyoshi *et al.* [40] characterized PERV subtypes in lymphocytes of miniature swine. Similarly, Bösch *et al.* [89] performed PCR screening for PERV *env*A, *env*B, and *env*C genes in a specific-pathogen-free Large White swine herd. Mang *et al.* [90] conducted studies on PERV subtype distribution and copy number estimation using nested PCR in five breeds of domestic pigs and wild boar. These studies indicate that primers complementary to *env* can be used to determine which PERV subtype is present in the test material, and thus, whether it is polytropic or ecotropic virus. They can also indicate whether more than one subtype is present, showing that recombination can occur between subtypes.

The third group consists of primers complementary to LTR sequences, which enable amplification of the entire PERV genome. This allows characterization of full-length proviral DNA, comparison with sequences obtained for other PERVs, and evaluation of the potential of particular PERVs to undergo recombination events [47,65,91,92]. Sypniewski *et al.* [47] and Machnik *et al.* [65] used long-range PCR to detect full-length PERV DNA and to discriminate them from defective sequences, PCR primer selection depends on the question to be answered. Only when PCR is carried out with the simultaneous use of primers from all of the groups is it possible to assess the ability of the virus to express functional viral RNA [65]. This possibility is afforded by multiplex PCR. The specificity of the PCR reaction can be achieved by appropriate primer design. The synthesis of nonspecific products can be reduced by using dual priming oligonucleotide (DPO) primers. DPO primers are constructed from two segments joined by a polydeoxyinosine [poly(I)] linker. The 5’ segment is 18–25 bases in length and preferentially binds to the template DNA, initiating stable annealing. The 3’ segment is 6–12 bases in length and selectively binds to its target, blocking nonspecific annealing. The poly(I) linker placed between these two fragments divides the primer into two functionally different regions previously discussed, blocking the extension of primers that have bound nonspecifically to template DNA [93]. Use of a DPO system in multiplex PCR enables the simultaneous detection and subtype determination of PERVs in a small sample in a rapid, sensitive, specific and economical manner, and due to the same detection efficiency in internal organs, such as hair roots, it can be used as a screening method for the detection of specific PERV subtypes in material derived from pigs [94].

Very often, the host genome contains fragments of PERV genomes that are not replication competent [58]. Machnik *et al.* [65] showed that most PERV proviral DNA in pig blood samples was significantly mutated. It was also determined that some animals in pig herds can be PERV transmitters or non-transmitters, based on their transmission to human cells *in vitro* [21,59,61,95]. Furthermore, Jung *et al.* [96] indicate that there are differences within and between pig breeds in terms of PERV insertion site, and they suggested that selecting replication-competent PERV-free pigs is suitable for xenotransplantation. Niebert and Tönjes [97] also showed that the presence of PERV can vary in every pig. These authors suggested that careful selection of donor animals allows for elimination of replication-competent PERV by conventional breeding. It is known that the activity of LTRs can also influence the transcription of replication competent PERV [98]. Scheef *et al.* [98] and Denner *et al.* [43] showed that LTRs can differ in the presence and number of 39-bp repeats (18 and 21 bp subrepeats) located in the enhancer element of the U3 region, which can influence strong promoter activity in various cell lines. Niebert and Tönjes [97] found a dominance of PERV with repeat elements in the U3 region of LTRs. Moreover, proviral LTR adaptation of transcriptional activity was observed by dynamic changes of the number in 39-bp repeats during serial virus passaging. Similarly, Wilson *et al.* [99] indicated that these 18- and 21-bp subrepeats containing binding sites for transcription factors can contribute to transcription activation. On the other hand, both authors also observed, in transient expression assays, that an increase in the copy number of repeated sequences did not always result in a concomitant increase in transcriptional activation, depending on the cell types [98,99]. Scobie *et al.* [62] indicated that in certain cells, some PERV-B isolates exhibit limited LTR transcriptional activity, possibly because of the reduced number of 39 bp enhancer repeats compared with replication-competent PERV clones. Furthermore, similar sequence multimerizations in the U3 region of the LTR can influence gene expression of murine leukemia viruses [100], feline leukemia viruses [101] and koala retroviruses [102]. Jung *et al.* [103] showed both variability in the number of repeats on the 5’ PERV LTR region and unmethylated CpG sites in the U3 and U5 regions, which can have an impact on virus transcription activation. For xenotransplantation, consistent methylation of the U3 region seems important and suggests host suppression of retrovirus expression [103]. In their next studies, the authors analyzed the promoter activity of various PERV LTR elements by the characterization of DNA methylation and their sequences [104,105]. These authors also indicated that the differences in DNA methylation of LTRs seem to be specific for the cell or tissue type [104]. However, the heavy methylation in the majority of PERV 5’ LTRs was only revealed in porcine tissues. In the PK15 cell line mainly sparsely methylated or nonmethylated proviruses were found [106]. Park * et al.* [107] and Ha * et al.* [108] revealed that besides DNA methylation, inhibition of histone acetylation may also reduce the promoter activity of PERV LTR elements. Using real-time reverse transcription polymerase chain reaction (RT-PCR) and sodium bisulfite genomic sequencing, Nakaya * et al.* [109] observed that epigenetic modification can also regulate human gene expression, such as human PERV-A receptor.

#### 2.1.2. Qualitative Analysis of PERV RNA

The RT-PCR technique is used to detect gene expression and to detect viral RNA. In this method, the initial material is mRNA, which is the product of an active proviral gene. The first stage of this reaction is catalyzed by RT, followed by PCR amplification of the transcribed product. The entire RT-PCR reaction can be carried out in a single vessel. Primers that are specific for specific PERV sequences are used for this purpose [49,84,88]. The RT-PCR reaction can also be conducted in two stages. The reaction to synthesize cDNA (reverse transcription) can be carried out using primers consisting of decamer thymidine oligonucleotides [85] or random hexaprimers [110]. In the next stage, the cDNA obtained is the template for the PCR reaction.

#### 2.1.3. Detection of PERV by Hybridization Methods

*In situ* hybridization (ISH) and fluorescent *in situ* hybridization (FISH) methods can be also useful for detecting both proviral DNA or viral RNA; these methods allow the detection of viral nucleic acids at the site of occurrence inside the cell [61,111]. Chung *et al.* [111] confirmed inhibition of PERV expression in porcine kidney cells (PK15 cell line) by efficient multitargeting of the RNA interference gene using RT-activity and FISH analyses. 

#### 2.1.4. Inhibition of PERV Expression by RNA Interference

Moreover, *in vitro* studies have shown that RNAi eliminates PERV particles with a high level of efficiency. For example, in human cells infected with PERVs and in porcine endothelial cells, siRNA exhibited the highest efficiency in locating encoding gag and pol proteins in the RNA region [112,113]. The natural consequence of inhibiting PERV expression by RNAi *in vitro* is the current attempts to create transgenic animals whose cells are capable of synthesizing siRNA [79,114,115].

#### 2.1.5. Quantitative Analysis of PERV DNA and RNA

Careful analysis of PERV DNA and RNA must not be restricted to qualitative analysis alone; it is important to gain information about the number of copies of DNA and RNA present. Quantitative PCR methods can be used to determine the number of copies of PERV provirus DNA, while real-time RT-PCR allows the determination of the level of RNA, which is a measure of DNA transcriptional activity. In these reactions, the amount of amplicon being synthesized is determined using fluorescent reporter molecules (fluorochromes); the most commonly used is fluorochrome SYBR Green I [48,50,51]. 

RNA or DNA can be quantified by either of two methods. The initial number of copies of RNA or DNA can be calculated by relating the PCR signal to a standard curve, where the amount is expressed as pg of RNA/DNA extract, or it can be expressed as a proportion of an endogenous control [66,87,88,116,117].

Endogenous control is achieved by using reference genes, which are usually genes required for the maintenance of basic cell function (housekeeping genes). The optimum reference gene should be transcribed permanently and at a constant level in all cells of the organism and at all times, and its transcription should not be dependent on internal or external factors [118]. Reference genes used in the analysis of the nucleic acids of porcine cells include: beta-actin (ACTB), beta-2-microglobulin (B2M), glyceraldehyde-3-phosphate dehydrogenase (GAPDH), hydroxymethylbilane synthase (HMBS), hypoxanthine phosphoribosyltransferase 1 (HPRT1), ribosomal protein L4 (RPL4), succinate dehydrogenase complex, subunit A (SDHA), TATA box binding protein (TBP), topoisomerase II beta (TOP2B) and eukaryotic translation elongation factor 1 alpha 1 (EEF1A1) [118,119]. The main reference genes used in PERV determinations are GAPDH, ACTB and HPRT [23,110,120]. It is necessary to check, each time, whether the reference gene selected can be used in the test system being studied. Among the genes that have been investigated, EEF1A1 is suitable, in terms of stability of expression, as a reference gene for normalization of mRNA expression in pigs [119]. The number of copies should also affect the endogenous control chosen. ACTB and RPL4 are the most appropriate for highly abundant transcripts, while TPB and HPRT1 are the most appropriate for less abundant transcripts [118].

These methods have a sensitivity that allows detection of as few as 1.1 × 10^3^ copies per genome by PCR, one copy per genome by nested PCR, and 100 molecules by real-time PCR. However, the use of methods with this level of sensitivity also poses the risk of contaminations and false positive results [121]. Therefore, PCR designed to detect proviruses or retrovirus infections should be performed in accordance with good laboratory practices. Recent reports clearly demonstrated that the detection of viruses in human tissues was the result of laboratory contaminations rather than true virus infection [122].

Ma *et al.* [50] found the upper limit of quantification by the SYBR Green I-based real-time PCR assay to be 8 × 10^6^ copies/μL, and that of conventional PCR to be 10^2^ copies/μL. SYBR Green I-based real-time PCR thus exhibits detection sensitivity as low as a single copy of the PERV genome per porcine genome, which is 100 times more sensitive than conventional PCR, making the SYBR Green I-based quantitative PCR assay a simple, sensitive, specific, reproducible, and rapid method of estimating copy numbers of PERV integrated into the host genome. These features make it an excellent tool for screening recipients of xenografts or source animals. 

One problem that has been noted in work on quantitative tests for the detection of hepatitis B virus DNA is that they are limited by a lack of standardization of the assays and that different assays have different sensitivities and ranges of linearity [123]. The same is true for tests that detect PERV DNA.

Quantitative analysis of RNA using the PCR technique is more complicated compared to the equivalent analysis for DNA [124]. Attention must be paid to the quality and quantity of RNA templates; standard internal controls must be used to monitor RT and PCR efficiencies, and the techniques and equipment used in different laboratories must be standardized. However, the main reasons for the greater degree of complication are difficulties in selecting an appropriate standard curve and problems with interpretation of the results obtained, such as expression of the results in terms of a comparison with the endogenous control or as an amount per 1 μg of total RNA extract [125].

Moreover, the expression of PERVs may be characterized by expression of both a full-length mRNA (encoding for Gag and Pol) and a spliced mRNA (encoding for Env). The expression of the spliced mRNA is a prerequisite for the translation of Env protein [52,67,126]. Karlas *et al.* [126] and Bittmann *et al.* [52] showed that primers specific to PCR assay detected both types of mRNA, which are complementary to sequences located in front of the splice donor, behind the splice donor, and in the *env* region behind the splice acceptor. The latter author noticed expression of both mRNAs in many pig organs, but at different levels. These authors also indicated that expression of spliced mRNA correlated with the expression of the full-length mRNA in various organs. Furthermore, different variants of spliced *env* mRNA were observed in PK15 cells [52]. The detection of spliced mRNA may be useful in evaluating the protein translation opportunity and viral particle release from cells.

Quantitative analysis of RNA can be used to perform PERV infectivity assays. In addition, greater knowledge can be gained by other methods, such as determination of RT activity.

#### 2.1.6. Determination of RT Activity

A finding of RT activity in culture supernatant or in plasma may indicate retroviral expression. In conjunction with an increase in the number of copies of PERV RNA, it is an indicator of active PERV replication and can be used to monitor the multiplication of retroviruses in cell cultures. The fact that RT activity is a marker of retroviruses in general means that a finding of RT activity alone may indicate the presence of other retroviruses in the sample. In turn, this means that its absence in patients’ sera can be treated as an indication of the absence of other, unrecognized retroviruses of porcine origin [127]. RT activity can be determined with the help of pre-prepared reagent kits [127,128] or by using real-time PCR [91,129].

### 2.2. Selection of Pigs that Exhibit Low-Level Expression of PERV-A and PERV-B

Pigs whose organs are to be used for xenotransplantation should have as low a level of PERV expression as possible [68]. The level of expression is measured following stimulation of PERV expression in pig PBMCs using mitogens such as phytohemagglutinin. Different pig breeds and different individuals of the same breed vary in their ability to produce PERV particles, and the release of PERV particles from PBMCs correlates with the extent of proliferation [46]. Pigs that exhibit low-level expression after such treatment are classified as low producers of PERVs. The level of expression determined using real-time RT-PCR has been related to expression of PERVs in the PERV-producing PK-15 cell line [120]. Using co-cultures of miniature swine PBMC with human and porcine cells has enabled the identification of “null” swine that do not transmit PERV to human and porcine cells; transmitters that can transmit recombinant PERV-A/C to human and pig cells; non-transmitters that cannot transmit PERV and non-recombinant PERV that only replicates in pig cells [61]. Wood *et al.* [61] indicated that the level of activity of PERV-C *loci* may influence the production of human-tropic, replication-competent recombinant PERV-A/C. In addition, Hector *et al.* [130] suggested that a lack of PERV-C active *loci* could reduce the risk of release of PERV-A/C recombinants.

### 2.3. Selection of Pigs That Do Not Contain PERV-C in Their Germ Line

Although it is not as common as the other two subtypes [120,131], the ability of PERV-C to recombine with PERV-A presents a particular problem, because the PERV-A/C recombinants are human-tropic and exhibit a greatly increased replication capacity, which may be associated with higher pathogenicity [38,42,95]. Besides the possibility of mutation in the C-terminal region of the surface protein SU [37,77], this subtype is highly undesirable as material for xenotransplantation. A recombination in which the region responsible for binding to the host cell receptor, particularly of human cells, comes from PERV-A and the remainder comes from PERV-C can cause the generation of a recombinant PERV-A/C virus able to infect human cells with an efficiency of around 500 times greater than PERV-A itself [42].

PERVs can be detected with PCR techniques that employ primers complementary to the PERV envelope gene *env*. The use of DPO primers, which have greater sensitivity, is also promising. The same efficiency in detecting PERV-C in hair roots as in internal organs offers hope for the development of rapid screening tests [94]. However, because of the possibility of contamination with cells from PERV-C-positive animals, it is recommended that blood samples be used for these tests [121]. Furthermore, because of the possibility of recombination and its possible consequences, if PERV-C virus is detected, appropriate primers can then be used in a further test, which will allow detection of recombinant PERV-A/C forms in the material being tested. These primers can be a combination of PERV-A *env* VRB forward and PERV-C TM reverse primers [63,120,132], PERV-A *env* VRB forward and PERV-C *env* reverse primers [63,120], or PERV-A *env* VRA forward and PERV-C TM reverse primers [132].

### 2.4. Screening Xenotransplant Recipients for PERV Transmission Using Assays to Differentiate Transmission and Chimerism

It is essential to be able to recognize microchimerism, or the long-term persistence of porcine cells in the blood or other tissues of xenotransplant recipients because this may lead to an erroneous finding of PERV transmission. Besides using molecular biology techniques to monitor recipients for the possibility of PERV transmission following xenotransplantation, immunochemical methods can also be used.

#### 2.4.1. Screening Xenotransplant Recipients—Molecular Biological Methods

All of the methods discussed in the section on careful screening of the source pig herd are used in monitoring recipients for the possibility of PERV transmission following xenotransplantation. Tests intended to detect provirus integrated into recipient DNA or the presence of retrovirus in the recipient’s blood are carried out on the sera or PBMCs of xenotransplant recipients. If a positive result is obtained, a confirmatory test needs to be carried out to determine whether the result was caused by a real infection or by the presence of porcine cells from the transplanted material in the sample. To do so, a PCR should be carried out on material from the donor pig for the presence of PERVs and a gene characteristic only of porcine cells. The results obtained should be compared with those from the same tests carried out on the sample from the recipient. A comparison of the results—the ratios of PERV DNA to pig cellular gene in the donor pig DNA and in the patient sample—indicates whether microchimerism has occurred or if there is a true PERV infection [5,133]. The porcine mitochondrial gene that encodes cytochrome B is a well-documented marker, used in phylogenetic tests, that allows detection of microchimerism following xenotransplantation [133]. The use of appropriate primers allows detection of one porcine PBMC cell in 10^6^ to 10^7^ human cells [23]. Using this approach, no evidence of PERV infection was found in 12 patients who had received porcine islet transplants [87,134].

#### 2.4.2. Screening Xenotransplant Recipients—Immunochemical Methods

Immunochemical methods require the use of purified proteins to detect antibodies and allow production of suitable antibodies to serve as a positive control. These proteins include the envelope proteins gp70 and p15E, the capsid protein, and whole virus lysate. Kaulitz *et al.* [135] induced neutralising antibodies specific for the surface envelope protein gp70 of PERV. However, in view of the fact that truncated gp70 showed improved purification efficiency than intact form, a specific anti-gp70 antibody was also developed by immunizing mice with a 42 kDa truncated protein of 357 amino acids, Env-aa357, expressed in an *E. coli* vector [136]. Peptides were synthesized that correspond to the immunosuppressive domain of the transmembrane protein p15E (amino acids 532–549), the predicted cysteine–cysteine loop of p15E (amino acids 544–562), and the cytoplasmic domain of PERV-A (amino acids 644–660). In addition, recombinant transmembrane protein rp15E was developed, which corresponds to the central domain of PERV-A p15E without the hydrophobic N-terminal fusion peptide or the C-terminal transmembrane and cytoplasmic domains (amino acids 488–597). Protein from whole virus was obtained following purification of a virus produced by porcine PK-15 cells by ultracentrifugation and pelleting through a sucrose gradient [137]. Recombinant Gag protein, and more precisely, the C-terminal half, is thought to be more antigenic and has been produced using recombinant DNA technology [138].

These proteins have been used to produce antibodies and to develop methods for the detection of antibodies in the sera of recipients. An example is the ELISA test for the detection of anti-PERV antibodies developed by Tacke *et al.* [137]. This test is based on synthetic peptides that correspond to specific regions of p15E, which are highly conserved in different retroviruses—the highly conserved immunosuppressive domain and the adjacent highly immunogenic and immunodominant domain. The method is based on one that was previously developed and used to detect human immunodeficiency virus type-1 (HIV-1) infections. Peptides were immobilized in 96‑well plates and used in an ELISA test to detect antibodies to specific PERV antigens. To confirm positive results, a Western blot technique was used with recombinant TM protein (rp15E), purified capsid protein (p27 Gag) and purified whole virus produced by HEK293 cells. A minimum of two reactions against different proteins (Gag and Env), were considered necessary to confirm infection, as is the case with verifying HIV infections [137]. This step is intended to eliminate false positive reactions caused by the presence of cross-reacting antibodies.

The anti-sera against PERV proteins and peptides used by Tacke *et al.* [137] were obtained by immunizing rabbits and goats with whole virus or isolated proteins, such as p27 Gag, and by immunization with synthetic peptides. Other synthetic antibodies include antibodies against PERV Env-aa357 and against recombinant Gag protein. Antibodies obtained against the Env-aa357 protein are specific to PERV Env and can detect both PERV-A and PERV-B envelope proteins [136]. Monoclonal antibodies against recombinant Gag protein are highly sensitive and specific to both the recombinant and native forms of the PERV Gag protein. They were developed against the C-terminal half of PERV Gag (Gag-CB7), which is thought to be the most antigenic. Their use may be a good solution in the search for PERVs in xenotransplantation patients and in biological material obtained from pigs [138].

In view of the current lack of a suitable animal model for evaluating cross-species PERV transmission, it is difficult to assess the efficacy of assays that could be used to monitor PERV transmission *in vivo* [68]. Therefore, when developing methods, it is important to consider the experience gained from infections with other retroviruses [5].

Interestingly, microarray analysis enables the detection of expression changes of several thousand genes simultaneously in response to different stimuli, including virus infection [139,140]. In previous research in the area of virology, microarray analysis was also used as a diagnostic tool to detect the presence of different viruses or to discover new viruses [141,142,143]. In the xenotransplantation context, Fishman *et al.* [144] suggested the possibility of using microarrays to detect various pathogens in xenograft recipients. In addition, previous research using the microarray technique has indicated an interaction between PERVs and human cells and suggested that PERV infection of human dermal fibroblasts can change the gene expression profile [145].

## 3. Closing Remarks

In summary, detecting PERVs in recipients of xenotransplantation products and/or source animals should be carried out using all available molecular methods in accordance with the International Xenotransplantation Association recommendations. This concept has enormous significance for the promotion of practices that aim to reduce the risk of transmitting PERV infection, and also for the development of more sensitive techniques for PERV detection, thus further contributing to improved xenotransplantation safety.
